# Early onset coats’ disease initially treated as unilateral ROP at 39 weeks postmenstrual age: a case report

**DOI:** 10.1186/s12886-017-0536-x

**Published:** 2017-08-16

**Authors:** Jie Peng, Qi Zhang, Chunli Chen, Qiujing Huang, Yian Li, Peiquan Zhao

**Affiliations:** 1 Department of Ophthalmology, Xin Hua Hospital Affiliated to Shanghai Jiao Tong University School of Medicine, Shanghai, 200092 China; 2 Department of Ophthalmology, Shengli Oilfield Central Hospital, Shangdong Province, China

**Keywords:** Coats’ disease, Retinopathy of prematurity, Exudative retinal detachment, Anti-VEGF, Laser photocoagulation

## Abstract

**Background:**

This is the youngest case of Coats’ disease, in terms of postmenstrual age (PMA), to be reported in the literature. This case highlights the remarkable variations in the clinical manifestations and the very early onset of Coats’ disease. This case is unusual in both the age of onset and atypical clinical features, which resemble retinopathy of prematurity (ROP).

**Case presentation:**

We report a case of a preterm boy born at 31 5/7 weeks gestational age who presented with atypical Coats’ disease and was initially diagnosed as having ROP of only one eye at 39 weeks PMA. After initial laser treatment, severe exudative retinal detachment (ERD) occurred after initial laser treatment for ROP. Fundus fluorescein angiography (FFA) showed telangiectasia and anastomosis of peripheral retinal vessels and nonperfusion areas, and the diagnosis of Coats’ disease was thus established. A series of intravitreal injections of ranibizumab (IVR) and laser ablations were performed to resolve the exudation and to ablate the abnormal vessels. At the last visit, the retinopathy was under control, and useful vision was preserved.

**Conclusions:**

Coats’ disease resembling stage 3 ROP can be detected before the expected date of childbirth. Therefore, asymmetric ROP should be differentiated from Coats’ disease.

## Background

Coats’ disease is an idiopathic retinal vasculopathy that is usually unilateral and sporadic and has no accurate genetic basis. It has a strong male predominance, and the mean age at diagnosis is 10 years [1]. Typical clinical manifestations of Coats’ disease are telangiectasias of peripheral retinal vessels with subretinal and intraretinal exudation. Laser ablations of the abnormal retinal vessels help to preserve vision in milder cases. Severe cases have been surgically treated [1]. Anti-VEGF (anti-vascular endothelial growth factor) drug is an adjuvant therapy and promotes the resolution of exudates and the regression of serous retinal detachment [2], but caution is advised.

Here, we report the youngest case of Coats’ disease in the literature in terms of PMA. This case highlights the remarkable variations in the clinical manifestations and very early onset of Coats’ disease. This case is unusual in both the age of onset and atypical clinical features resembling ROP. The study was approved by Ethical Committee of Xinhua Hospital at the Shanghai Jiao Tong University School of Medicine and was conducted in accordance with the principles of the Declaration of Helsinki.

## Case presentation

**Fig. 1:**
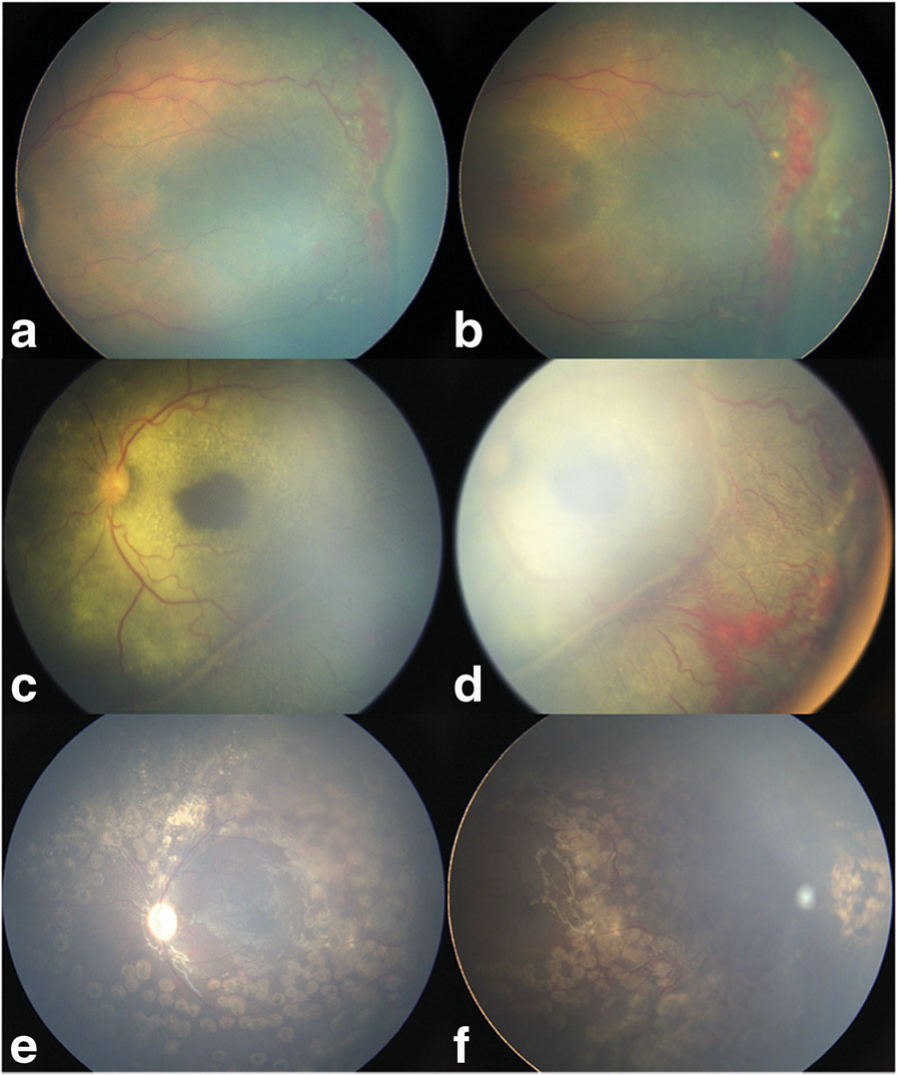
Retcam (Clarity Medical Systems, Pleasanton CA) images of the *left eye*. **a**. At 39 weeks’ PMA, tortuous and dilated vessel, temporal ridge of abnormal vessels in Zone II and avascular zone in the periphery were detected. **b**. One week after laser photocoagulation at 40 weeks’ PMA, ERD occurred. **c**. and **d**. One month after laser ablation at 43 weeks’ PMA, ERD and retinal vasculopathy aggressively progressed. **e**. and **f**. Last visit at 1 year after presentation, conditions were controlled and useful vision was preserved

A Chinese male infant who weighed 1650 g was born at 31 5/7 weeks gestation via vaginal delivery. The boy stayed in the NICU for 1 week and oxygen inhalation was given to him for 3 days. The boy did not come for the screening visit until 39 weeks PMA due to the guardian’s reason. At first fundus screening at 39 weeks PMA, he was initially diagnosed with stage 3 ROP in zone II plus another disease in a different hospital (Fig. 1a). The optic nerve of the patient was normal. Immediate laser ablation was performed in the other hospital (laser wavelength: 532 nm, power: 200 mV, total number of spots: 1024 spots). ERD was observed after 1 week (Fig. 1b), followed by aggressive progression during a later month. The boy was transferred to our department. Upon examination, the left eye had normal anterior segment, yellow subretinal exudates and partial ERD, as well as extensive abnormal retinal vessels (Fig. 1c and d). The right eye was normal upon examination.

**Fig. 2:**
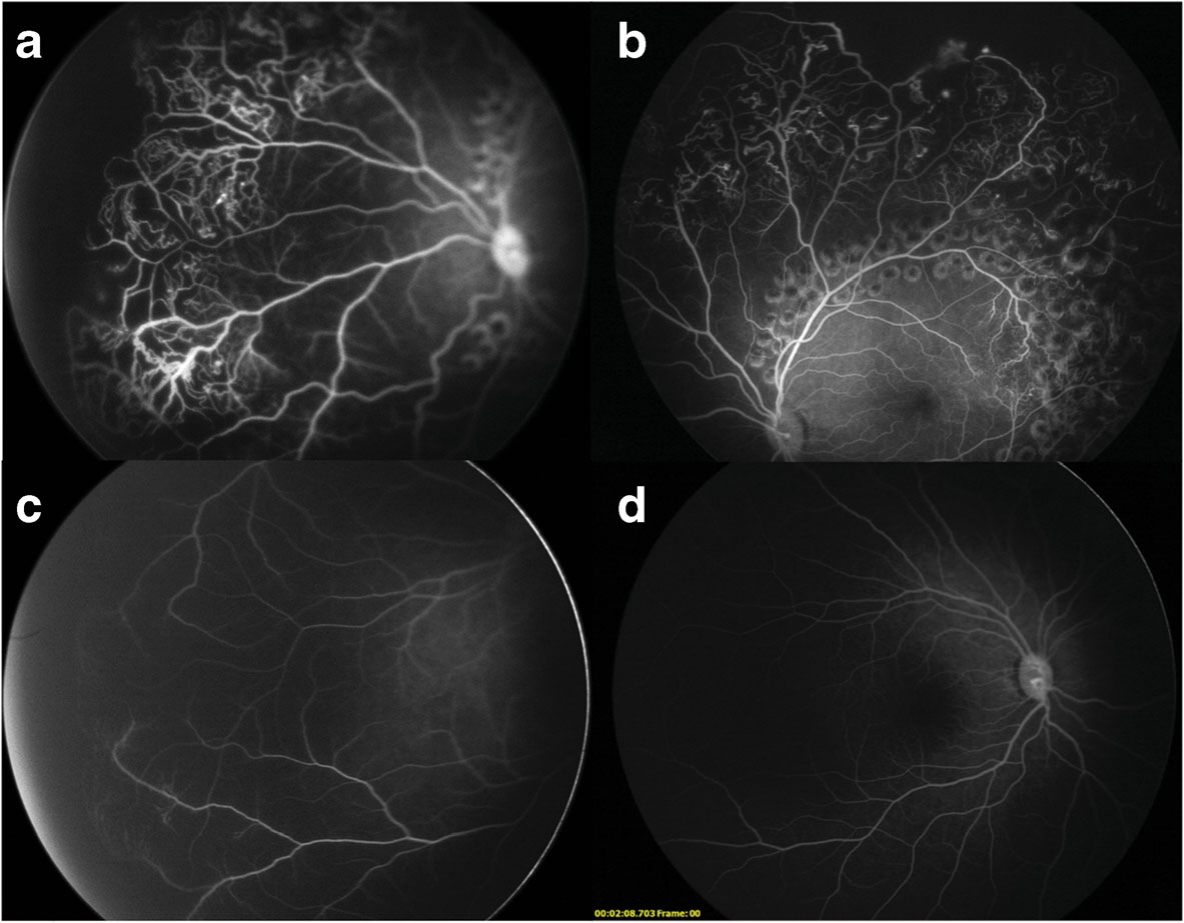
FFA images at 58 5/7 weeks’ PMA. FFA showed telangiectasias, microaneurysms and nonperfusion areas of the *left eye* and temporal nonperfusion area in the *right eye*. **a**. Nasal field of the *left eye*. **b**. Superior field of the *left eye*. **c**. Temporal field of the *right eye*. **d**. Posterior pole of the *right eye*

Treatment was in favor of immediate IVR (0.25 mg/0.025 ml) at 43 weeks PMA. All of the treatments were approved by the patient’s guardians via written consent. Twenty-five days after IVR, the vasculopathy and ERD almost regressed. We first tried traditional trans-pupillary laser photocoagulation. However, it was ineffective because of the remaining ERD. Endolaser photocoagulation by a two-port pars plana nonvitrectomy approach [3] combined with IVR was performed at 46 5/7 weeks PMA. During the intraocular treatment, telangiectasias of the peripheral retinal vessels and subretinal exudation were noted, indicating Coats’ disease (Stage 3A). FFA at 58 5/7 weeks PMA showed telangiectasias, microaneurysms and anastomosis of peripheral retinal vessels and nonperfusion areas and laser spots in all quadrants (Fig. 2a and b). Only a few subretinal exudations without ERD were noted because of the treatment with IVR. Abnormal vessels and a nonperfusion area (NPA) were detected in the periphery of the right eye (Fig. 2c), which was missed by the previous traditional fundoscopy. Due to the bilateral changes, FFA of the parents and a genetic analysis (Panel E, MyGenostics, Beijing, China) for familiar exudative vitreoretinopathy (FEVR) were performed for this family. All of the results turned were normal, and FEVR was thus excluded.

The clinical and FFA findings contributed to the diagnosis of Coats’ disease. A series of IVRs and laser ablations were performed to resolve the exudation and ablate the abnormal vessels. The first IVR was performed at 43 1/7 weeks PMA, followed by endolaser photocoagulation combined with IVR at 46 5/7 weeks PMA, trans-pupillary laser photocoagulation (TPLP) combined with IVR at 58 5/7 weeks PMA, TPLP at 60 5/7 weeks PMA, and endolaser photocoagulation combined with IVR at 67 6/7 weeks PMA. At the last follow-up, which was 1 year after presentation, the left eye had much better anatomical outcomes and useful vision. Fundus images of the left eye showed a normal macular, flat retina without telangiectasias or exudation (Fig. 1e and f). The right eye remained stable and healthy with traditional fundoscopy. The boy could grab a green bean that was 33 cm away using vision from his left eye. However, the visual acuity was not assessed due to patient’s poor cooperation.

## Discussion and conclusions

A 39-week PMA preterm boy presented with atypical Coats’ disease and was initially diagnosed as having ROP of only one eye. Laser ablation was initially performed, and the eye developed ERD that was associated with massive retinal vasculopathy afterwards, which implied Coats’ disease. Intravitreal injections of anti-VEGF drugs and laser ablations were given to preserve vision of this eye.

Although uncommon, ERD after conventional laser therapy for ROP has been reported [4], which makes it difficult to differentiate Coats’ disease from ROP. However, ROP tends to be symmetric. Asymmetric ROP is uncommon and may be associated with optic nerve hypoplasia [5] or peripapillary staphyloma [6]. In this case, no optic disc anomaly was detected. Therefore, the diagnosis of asymmetric ROP should be performed with caution. The ERD presented in this case may be caused by laser-induced damage and the retinal vasculopathy itself. Coats’ disease is a rare idiopathic retinal vasculopathy. The typical clinical manifestations of Coats’ disease are telangiectasias of peripheral retinal vessels with subretinal exudation and intraretinal exudation [1]. FFA helps to differentiate Coats’ disease from other diseases. FFA of this case showed telangiectasias, microaneurysms and nonperfusion areas of the left eye and a temporal nonperfusion area of the right eye. FEVR was excluded from the negative familial history and from the results of the genetic analysis. All of the results implied a diagnosis of Coats’ disease. The diagnosis of asymmetric ROP should be made with caution and after ruling out other ocular anomalies, such as optic disc anomalies and Coats’ disease.

Exudative retinal detachments that are caused by Coats’ disease or ROP responded favorably to intravitreal anti-VEGF injections [2, 4]. IVR combined with laser ablation may be the best choice for treatment. In eyes with serous retinal detachment in which telangiectasias cannot be reached by regular laser ablation, endolaser photocoagulation via a two-port pars plana nonvitrectomy approach is an effective treatment for advanced Coats’ disease with ERD [3]. This procedure could be more thorough and efficient and may reduce the number of treatment sessions. In total, four sessions of IVR, two sessions of endolaser photocoagulation via a two-port pars plana nonvitrectomy approach, and two sessions of TPLP were performed to cure the disease.

In this case, abnormal vessels combined with a nonperfusion area were seen in the right eye (Fig. 2c). Peripheral retinal nonperfusion do exist in fellow eye in Coats’ disease [7]. Bilateral vascular abnormalities are more common than what was previously reported. This manifestation supports a systemic and/or genetic association with Coats’ disease.

This case is unusual in both the age of onset and the atypical clinical features resembling ROP. A clinical manifestation of a ridge of abnormal vessels at the onset of disease has never been reported in Coasts’ disease. To our knowledge, there are only three cases of premature babies with Coats’ disease that have been previously described in the literature, and those with Coats’ disease who have xantocoria present with a more aggressive course of disease and with a poorer visual acuity [8–10]. Furthermore, this is the youngest case of Coats’ disease with rapid progression to exudative retinal detachment after conventional laser ablation reported in the literature in terms of PMA. However, the visual acuity was not assessed due to the patient’s poor cooperation, but this should be observed in the near future.

In conclusion, this case highlights the remarkable diversity in the clinical presentation and morphology of Coats’ disease, a very early onset of Coats’ disease and the importance in differentiating it from other retinal vascular diseases such as ROP. Coats’ disease can occur even before the expected date of birth. Its early treatment allows for the maintenance of an acceptable visual acuity. Anti-VEGF therapy combined with laser ablations may be effective treatments. When shallow retinal detachment occurred, endolaser photocoagulation via a two-port pars plana nonvitrectomy approach is an effective method.

## Abbreviations

ERD: Exudative retinal detachment
FEVR: Familiar exudative vitreoretinopathy
FFA: Fundus fluorescein angiography
IVR: Intravitreal injection of ranibizumab
PMA: Postmenstrual age
ROP: Retinopathy of prematurity
VEGF: Vascular endothelial growth factor

## References

[1] Shields JA, Shields CL (2002). Review: coats disease: the 2001 LuEsther T. Mertz lecture Retina.

[2] Sigler EJ, Randolph JC, Calzada JI, Wilson MW, Haik BG (2014). Current management of coats disease. Surv Ophthalmol.

[3] Cai X, Zhao P, Zhang Q, Jin H (2015). Treatment of stage 3 Coats’ disease by endolaser photocoagulation via a two-port pars plana nonvitrectomy approach. Graefes Arch Klin Exp Ophthalmol = Albrecht von Graefes Archiv fur klinische und experimentelle Ophthalmologie.

[4] Ehmann D, Greve M (2014). Intravitreal bevacizumab for exudative retinal detachment post laser therapy for retinopathy of prematurity. Can J Ophthalmol.

[5] Arnold RW (2008). Optic nerve hypoplasia potentiates retinopathy of prematurity. J Pediatr Ophthalmol Strabismus.

[6] Kim BM, Shapiro MJ, Miller MT, Blair MP (2011). Peripapillary staphyloma with associated retinopathy of prematurity. Retinal Cases Brief Rep.

[7] Blair MP, Ulrich JN, Elizabeth Hartnett M, Shapiro MJ (2013). Peripheral retinal nonperfusion in fellow eyes in coats disease. Retina.

[8] Maruoka K, Yamamoto M, Fujita H, Tahara Y, Ishibashi T (2005). A case of coats’ disease in a low-birth-weight infant. Ophthalmol J Intern D'ophtalmol Intern J Ophthalmol Zeitschrift fur Augenheilkunde.

[9] Song HP, Ai H, Zhu Q, Lei CL, Wang JZ, Lei XQ (2012). Stage 3B coats disease in a premature and low-birth-weight infant. Chinese Med J.

[10] Gursoy H, Erol N, Bilgec MD, Basmak H, Kutlay O, Aslan H (2015). Bilateral Coats’ disease combined with retinopathy of prematurity. Case Rep ophthalmol Med.

